# The sedoheptulose kinase CARKL controls T-cell cytokine outputs and migration by promoting metabolic reprogramming

**DOI:** 10.1093/discim/kyae016

**Published:** 2024-11-19

**Authors:** Michelangelo Certo, Jennifer Niven, Robert Haas, Paula Rudzinska, Joanne Smith, Danilo Cucchi, Jose R Hombrebueno, Claudio Mauro

**Affiliations:** College of Medicine and Health, University of Birmingham, Birmingham, GB, UK; William Harvey Research Institute, Barts and The London School of Medicine and Dentistry, Queen Mary University of London, London, GB, UK; College of Medicine and Health, University of Birmingham, Birmingham, GB, UK; William Harvey Research Institute, Barts and The London School of Medicine and Dentistry, Queen Mary University of London, London, GB, UK; College of Medicine and Health, University of Birmingham, Birmingham, GB, UK; William Harvey Research Institute, Barts and The London School of Medicine and Dentistry, Queen Mary University of London, London, GB, UK; William Harvey Research Institute, Barts and The London School of Medicine and Dentistry, Queen Mary University of London, London, GB, UK; College of Medicine and Health, University of Birmingham, Birmingham, GB, UK; College of Medicine and Health, University of Birmingham, Birmingham, GB, UK; William Harvey Research Institute, Barts and The London School of Medicine and Dentistry, Queen Mary University of London, London, GB, UK

**Keywords:** CARKL, T cells, pentose phosphate pathway, reprogramming, immunometabolism, inflammation

## Abstract

**Background:**

Immunometabolism is a crucial determinant of immune cell function, influencing cellular activation and differentiation through metabolic pathways. The intricate interplay between metabolism and immune responses is highlighted by the distinct metabolic programs utilized by immune cells to support their functions. Of particular interest is the pentose phosphate pathway (PPP), a key metabolic pathway branching out of glycolysis that plays a pivotal role in generating NADPH and pentose sugars crucial for antioxidant defense and biosynthesis. The sedoheptulose kinase Carbohydrate Kinase-like protein (CARKL), an enzyme involved in the PPP, emerges as a critical regulator of cell metabolism and was previously shown to play a role in macrophage function.

**Methods:**

This study delves into the impact of CARKL expression on T-cell functionality, revealing dynamic alterations in response to cellular activation. Notably, CARKL overexpression leads to significant metabolic shifts in T cells, affecting mitochondrial respiration, ATP production, and inflammatory cytokine profiles. Furthermore, CARKL modulation influences T-cell motility by regulating chemokine receptor expression, particularly compromising CXCR3 expression and impairing T-cell migration in response to specific chemokine signals.

**Conclusions:**

These findings underscore the multifaceted role of CARKL as a metabolic regulator shaping T-cell responses. Overall, our data reveal the complex regulatory mechanisms orchestrated by CARKL in T-cell function, with implications for immune regulation. Further exploration of the molecular interactions between CARKL and metabolic reprogramming in T cells could provide valuable insights into immune regulation and potential therapeutic strategies.

## Graphical Abstract

**Graphical Abstract:**
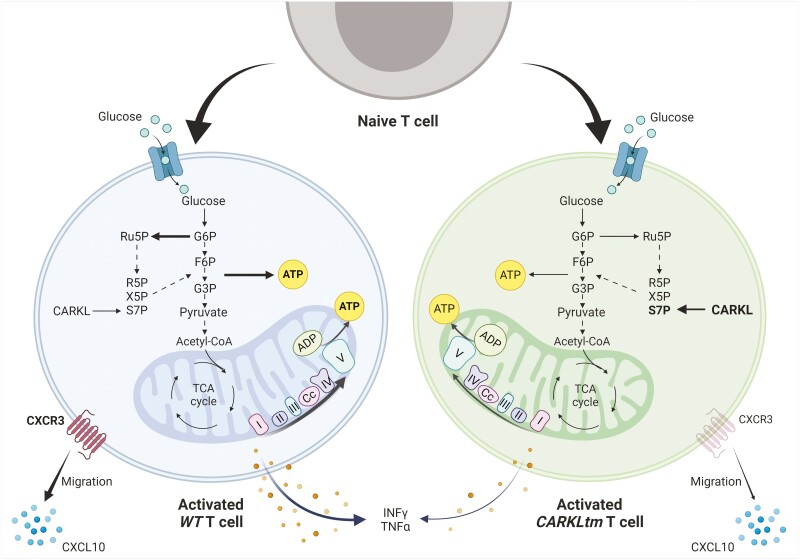

## Introduction

Immunometabolism plays a critical role in regulating immune cell function by influencing metabolic pathways that impact immune cell activation and differentiation. Immune cells utilize distinct metabolic programs to support their various functions, with shifts in metabolism dictating their responses to stimuli [1–3]. For instance, activated effector T cells rely on glycolysis and mitochondrial metabolism to meet their energetic demands for proliferation and cytokine production [4]. Conversely, regulatory T cells exhibit a preference for fatty acid oxidation, promoting their suppressive functions [5]. This metabolic flexibility allows immune cells to adapt to diverse microenvironments and immune challenges, highlighting the integral role of immunometabolism in shaping immune responses. Metabolic reprogramming enables effector immune cells to generate energy and biosynthetic precursors for cytokine production and cytotoxic activities. Moreover, nutrient availability influences immune cell metabolism and function, with specific nutrients like glucose, amino acids, and fatty acids serving as metabolic substrates that regulate immune responses [6]. The interaction between nutrient sensing and immune signaling is crucial in regulating immune responses, balancing immune activation (to fight infections) and immune tolerance (to prevent excessive or harmful immune reactions). The role of immunometabolism in immune response is still not fully understood, reflecting the complexity of metabolic interactions within the immune system. While studies have elucidated key metabolic pathways in immune cells that can be targeted to rephase the immune response, much less is known about the contribution of PPP in immune cell [7].

PPP is a crucial metabolic pathway that branches out of glycolysis and plays a key role in generating NADPH and pentose sugars. NADPH is essential for antioxidant defense and biosynthetic processes, while pentose sugars are important for nucleotide synthesis and DNA repair mechanisms [8]. In addition to these fundamental roles in cellular metabolism, emerging research has highlighted the significance of the PPP in modulating immune responses [8, 9]. Immunometabolism studies have shown that the PPP can directly impact the immune response by regulating the redox balance and the production of metabolic intermediates that influence immune cell function [10]. Indeed, immune cells, particularly activated T cells, rely on NADPH to support reactive oxygen species (ROS) production for antimicrobial defense and signaling processes [11]. PPP provides a major source of NADPH through the generation of reducing equivalents during the oxidative phase [8]. Manipulating PPP can therefore have profound effects on immune cell function and immune responses. Modulation of key enzymes in the PPP, such as glucose-6-phosphate dehydrogenase (G6PD), can alter NADPH production and redox balance in immune cells, thereby impacting their effector functions [12]. Pharmacological inhibitors or activators targeting enzymes in the PPP have the potential to reshape the immune response by modulating the metabolic state of immune cells. For example, inhibiting G6PD activity in activated T cells has been shown to impair their proliferation and effector functions, leading to reduced cytokine production and cytotoxicity [9]. Conversely, enhancing PPP flux through activation of G6PD or other PPP enzymes could promote NADPH production and support the metabolic demands of immune cells during infection or inflammation [12, 13]. Targeting enzymes of the PPP to rebalance the metabolic profile or redox state of immune cells may offer new therapeutic opportunities for many diseases.

CARKL, also known as sedoheptulose kinase (SHPK), is a relatively understudied yet intriguing enzyme that plays a crucial role in cellular metabolism. This enzyme is a carbohydrate kinase, catalyzing the conversion of sedoheptulose into sedoheptulose-7-phosphate, an intermediate of the PPP [14–16]. Some studies have highlighted the involvement of CARKL in immune cell function and inflammation [14, 17, 18]. It has been shown that CARKL can modulate LPS-induced cytokine production in macrophages [14]. Interestingly, the constitutive expression of CARKL in macrophages resulted in a reduction in glycolytic flux following LPS exposure, concomitant with an increase in oxygen consumption rate, thus resembling the metabolic signature typically associated with M2-like cells [14]. Conversely, when CARKL expression was knocked out, macrophages exhibited a metabolic shift towards an M1-like state (M1). Furthermore, the researchers discovered that the modulation of CARKL expression had a direct impact on the flow of glycolytic intermediates into the oxidative arm of the PPP. Specifically, overexpression and knockdown of CARKL led to a reduction and increase, respectively, in the redirection of metabolites towards the oxidative PPP by regulating the levels of sedoheptulose-7-phosphate [14]. Furthermore, it has more recently been reported that overexpression of CARKL suppresses the mRNA expression of G6PD, the key enzyme in the PPP. This overexpression also leads to a marked reduction in cell proliferation, ROS production, and IL1β secretion [18]. CARKL expression has more recently been associated with glioblastoma progression, with elevated expression levels associated with a worse prognosis [17].

Our study brings to light the role of CARKL as a key metabolic regulator in orchestrating T-cell responses.

## Materials and methods

### Mice

The mouse model used in our study is a transgenic model. SHPK transgenic mice were generated and generously provided by Arvand Haschemi from the Medical University of Vienna. In brief, the coding sequence for mouse Shpk mRNA (NM_029031.3) was cloned into the pCAGGS plasmid (Niwa *et al*., Gene (1991), 193–200 [PMID: 1660837], GenBank: LT727518.1). This plasmid was obtained from the BCCM/LMBP Plasmid Collection, and the integrity of the sequence was confirmed through DNA sequencing. The transgene, which includes regulatory elements, was constructed using Sal I and Hind III restriction sites, resulting in a total size of 3.7 kB. Transgenic animals were successfully produced with the assistance of Thomas Rülicke from the University of Veterinary Medicine Vienna and Biomodels Austria through pronuclear microinjection of the transgene into fertilized C57BL/6N mouse embryos. Mice were housed in a pathogen-free environment and kept under standard conditions with a 12-h day/night cycle with access to food and water ad libitum. Environmental enrichments were added to all cages. Genotyping of ear notches taken at weaning was performed by Transnetyx.

### Isolation and activation of CD4 T cells from murine spleen and lymph nodes

Murine spleens and lymph nodes were mechanically dissociated and filtered through a 70-µm cell strainer to obtain a single-cell suspension. The spleen cell suspension was then treated with red blood cell lysis buffer (eBioscience) for 5 minutes to remove red blood cells, followed by a wash with PBS. CD4 T cells were isolated using the Stemcell Technologies CD4 T Cell Isolation Kit, following the manufacturer’s instructions. The isolated cells were counted and resuspended in complete RPMI 1640 medium (Gibco), supplemented with 10% fetal bovine serum, 1% penicillin-streptomycin, 50 µM 2-mercaptoethanol, and 20 IU/ml IL-2 (PeproTech). Cells were plated at a density of 1 million cells per well in six-well plates. For activation, CD4 T cells were stimulated with plate-bound anti-CD3 (2 µg/ml, eBioscience) and soluble anti-CD28 (1 µg/ml, eBioscience). The cells were incubated at 37°C with 5% CO2. Activation times varied according to experimental requirements, with time points of 24-, 48-, and 72-hour post-activation.

### Seahorse metabolic flux assays

A Seahorse XFe96 Bioanalyzer (Agilent) was used to determine OCR and ECAR for sorted CD4 T-cell subsets. Cells were washed once and incubated in XF Seahorse RPMI Medium (Agilent) supplemented with 10 mM glucose, 1 mM sodium pyruvate, and 2 mM L-glutamine for the Mito Stress Test and the ATP Rate Assay, or with 2 mM L-glutamine for the Glycolysis Stress Test. Cells were seeded at 200,000 cells/well in Agilent Seahorse XFe96 cell culture microplates coated with Cell-Tak solution (Corning). Cells were equilibrated in a non-CO^2^ incubator for 1 hour prior to the Seahorse assay to allow for proper CO^2^ and temperature control.

The cellular bioenergetic profiles were measured by serial injections of specific drugs, based on the assay (see Table 1). Metabolic parameters were obtained from the XF Wave software (Agilent/Seahorse Biosciences) and calculated using Microsoft Excel.

**Table 1:** drugs used for the metabolic flux assays

Glycolysis Stress Test		
Drug	Volume	Concentration
Glucose	20 µl	10 µM
Oligomycin	22 µl	1 µM
2-DG	25 µl	50 mM
Mito Stress Test		
**Drug**	**Volume**	**Concentration**
Oligomycin	20 µl	1 µM
FCCP	22 µl	1.5 µM
Rotenone/Antimycin A	24 µl	1 µM
ATP Rate Assay		
**Drug**	**Volume**	**Concentration**
Oligomycin	25 µl	1.5 µM
Rotenone/Antimycin A	25 µl	1 µM

### RNA isolation, reverse transcription, and qRT-PCR

Total RNA was isolated from 1 × 10^6^ CD4 T cells using RNeasy Mini kit (Qiagen) following the manufacturer’s instructions and assessed for quality and quantity with a NanoDrop spectrophotometer using absorption ratios of 260/280 nm and 260/230 nm. Cells were lysed in RLT lysis buffer and nucleic acids were precipitated with 70% ethanol and RNA bound to spin columns. Following several washing steps, RNA was eluted in dH2O. Reverse transcription was performed using a high-capacity cDNA Reverse Transcription Kit (Applied Biosystems). Briefly, 1 μg of total RNA was reverse-transcribed into cDNA in a reaction mixture containing reverse transcriptase, random primers, and dNTPs. The reverse transcription reaction was carried out at 25°C for 10 minutes, followed by 37°C for 120 minutes, and 85°C for 5 minutes.

Gene expression analysis was performed using quantitative real-time polymerase chain reaction (qRT-PCR) with gene-specific primers and SYBR Green detection (BioRad). The qRT-PCR reactions were set up in triplicate in a 96-well plate using a real-time PCR system. Each reaction consisted of cDNA template, gene-specific primers (see Table 2), and SYBR Green master mix. The qRT-PCR cycling conditions were as follows: initial denaturation at 95°C for 5 minutes, followed by 40 cycles of denaturation at 95°C for 10 seconds, annealing at 60°C for 10 seconds, and extension at 72°C for 30 seconds. Melting curve analysis was performed to confirm the specificity of the PCR products.

**Table 2:** RT-qPCR primer sequences

Gene	Fwd primer sequence	Rev primer sequence
*Carkl*	CAGGCCAAGGCTGTGAAT	GCCAGCTGCATCATAGGACT
*Glut1*	CACTGTGGTGTCGCTGTTTG	ATGGAATAGGACCAGGGCCT
*Glut2*	GGAAGTCAGGGCAAAGAAAAGC	AATTGGCATCCGTGAAGAGC
*Glut3*	TGTCACAGGAGAAGCAGGTG	GCTCCAATCGTGGCATAGAT
*Glut4*	CTGCCCGAAAGAGTCTAAAGC	CAGCTCCTATGGTGGCGTAG
*Ifng*	ATCAGGCCATCAGCAACAAC	TGCATCCTTTTTCGCCTTGC
*Tnf*	TCGTAGCAAACCACCAAGTG	TTTGAGATCCATGCCGTTGG
*Il1b*	TGGACCTTCCAGGATGAGGACA	GTTCATCTCGGAGCCTGTAGTG
*Il17*	AAAGCTCAGCGTGTCCAAAC	TTCTGGAGCTCACTTTTGCG
*Il4*	TCGGCATTTTGAACGAGGTC	TGGTGTTCTTCGTTGCTGTG
*Il5*	CCGCCAAAAAGAGAAGTGTGG	TTCCATTGCCCACTCTGTACTC
*Il10*	AAACAAAGGACCAGCTGGAC	TTCCGATAAGGCTTGGCAAC
*Il13*	ATTGCATGGCCTCTGTAACC	GGCGAAACAGTTGCTTTGTG
*Cxcr3*	GCTCTTTGCCCTCCCAGATT	CAGCAGGAAACCAGCCACTA
*Ccr4*	GGACTAGGTCTGTGCAAGATCG	TGCCTTCAAGGAGAATACCGCG
*CCR7*	AGAGGCTCAAGACCATGACGGA	TCCAGGACTTGGCTTCGCTGTA

Relative gene expression levels were calculated using the 2^-ΔΔCt method, with normalization to a housekeeping gene (β-actin). Data were presented as fold change in gene expression compared to the control group. Statistical analysis was performed to compare gene expression levels between experimental groups.

### Western blotting

CD4 T cells were lysed in RIPA buffer containing protease inhibitors to extract total protein. Protein concentration was determined using the Pierce BCA Protein Assay kit (ThermoFisher #23225). Equal amounts of protein from each sample were mixed with Laemmli sample buffer and denatured by boiling at 95°C for 5 minutes. Protein samples were separated on Tris-glycine gels (Bio-Rad) and Tris-glycine SDS running buffer (Geneflow # B9-0034). After electrophoresis, proteins were transferred onto Bio-Rad Trans-Blot polyvinylidene difluoride (PVDF) membranes (Bio-Rad, 1704157) using Bio-Rad Trans-Blot Turbo transfer system.

The membranes were blocked in 5% nonfat milk or BSA in TBS-T buffer for 1 hour at room temperature to prevent nonspecific binding. The membranes were then incubated with primary antibody against the target protein (see Table 3) overnight at 4°C with gentle agitation. Following primary antibody incubation, the membranes were washed with TBS-T and incubated with HRP-conjugated secondary antibody (Cell Signaling Technologies) for 1 hour at room temperature. The membranes were washed again to remove excess secondary antibody. Protein bands were detected using Clarity Enhanced Chemiluminescence substrate (Bio-Rad #1705061) and visualized using the ChemiDoc MP Imaging System (Bio-Rad). ImageJ Fiji was used to quantify the intensity of protein bands. Beta-actin or GAPDH were used as loading controls for normalization.

**Table 3:** antibodies used for Western blotting

Protein	Supplier	Cat #	Host species	Molecular weight	Dilution
ACAD9	Cell Signalling Technology	9796	Rabbit	69 kDa	1:1000
CARKL	ThermoFisher Scientific	PA5-101269	Rabbit	52 kDa	1:500
CPT1A	Cell Signalling Technology	12252	Rabbit	88 kDa	1:1000
Fatty Acid Synthase	Cell Signalling Technology	3180	Rabbit	273 kDa	1:1000
Hexokinase I	Cell Signalling Technology	2824	Rabbit	102 kDa	1:1000
Hexokinase II	Cell Signalling Technology	2867	Rabbit	102 kDa	1:1000
Phosphofructokinase	Cell Signalling Technology	8164	Rabbit	80 kDa	1:1000
TOMM20	Sigma	HPA011562	Rabbit	16 kDa	1:1000
β-Actin	Cell Signalling Technology	4967	Rabbit	45 kDa	1:1000
GAPDH	Abcam	ab9485	Rabbit	37 kDa	1:1000

### Cytokine detection

For the detection of the cytokines, cytometric bead array (CBA) assay was performed using BioLegend LEGENDplex Mouse Th Cytokine Panel (BioLegend, catalog no. 741044) following the manufacturer’s recommendations, on a BD Fortessa flow cytometer (BD Biosciences) and analyzed using the LEGENDplex Data Analysis Software Suite.

### Transwell migration assay

Chemotaxis assays were performed using a 24-well Transwell system with 5 μm pore size inserts (Corning). T cells were resuspended in serum-free medium and 3 × 10^5^ cells were placed in the upper chamber of the Transwell insert, while the chemoattractant (CXCL10, 300 ng/ml; CCL22, 200 ng/ml; CCL19/21, 200 ng/ml, PeproTech) was added to the lower chamber.

The Transwell plate was incubated at 37°C in a humidified 5% CO^2^ incubator for 4 hours to allow T cells to migrate towards the chemoattractant. Control wells without chemoattractant were included to assess basal migration.

After the migration period, non-migrating cells in the upper chamber of the Transwell insert were removed, and migrating cells in the lower chamber were counted, and then the percentage of migrated cells was calculated.

### *In vivo* peritoneal recruitment model

*In vitro* activated WT or CARKLtm CD4 T cells were labeled with 7-hydroxy-9H-(1,3-dichloro-9,9-dimethylacridin2-one) (DDAO; Invitrogen) or carboxyfluorescein succinimidyl ester (CFSE; Abcam), respectively, and intravenously co-injected in recipient syngeneic female C57BL/6 mice (5 × 10^6^ cells/mouse) that had 3 hours prior received an intraperitoneal injection with CXCL10 (1200 ng/mouse) (*n* = 6 animals per genotype). Twenty-four hours later, peritoneal lavage, spleen, mesenteric, and peripheral lymph nodes were collected. T cells were stained for surface markers CD4 (RM4-5, ebiosciences) and CXCR3 (ebiosciences). Cells were acquired on an LSR Fortessa (BD Bioscience). All flow cytometry analysis were performed using Flow Jo Version 10 software (Treestar).

### Statistical analysis

Statistical details of experiments can be found in the figure legends. All data are expressed as ± SD as indicated in figure legends. All statistical analyses were carried out in GraphPad Prism 10. Significant differences are indicated as follows: *P ≤ 0.05, **P ≤ 0.01, ***P ≤ 0.001, ****P ≤ 0.0001.

## Results

### CD4 T-cell activation is associated with a reduction in CARKL expression

**Figure 1.**
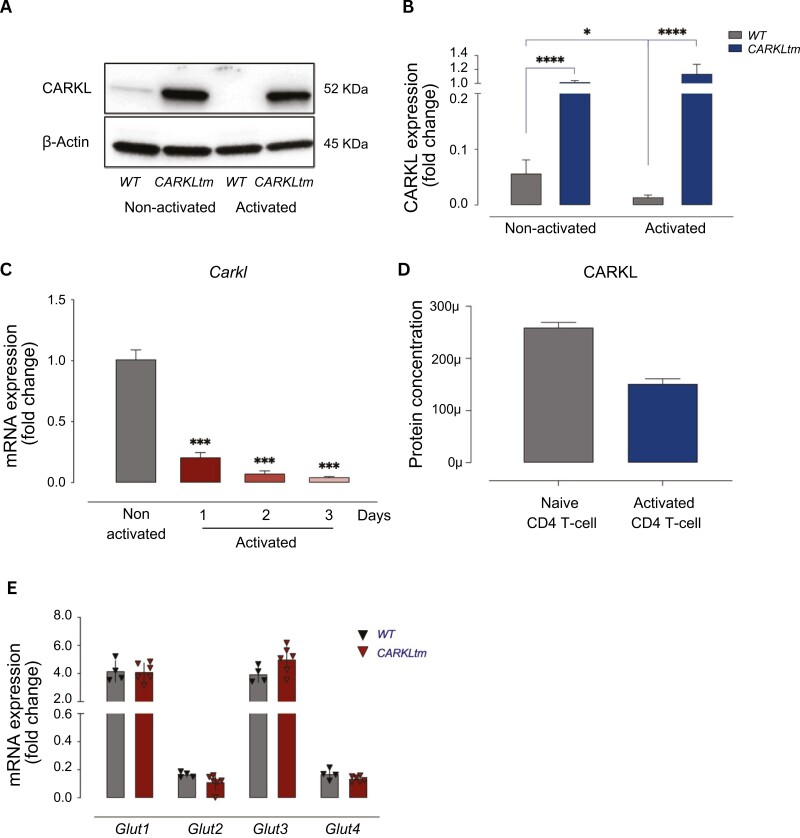
**Differential CARKL expression patterns in CD4 T cells from WT and CARKLtm mice. (A)** Immunoblot showing CARKL and β-actin loading control protein levels in non-activated and activated WT and CARKLtm CD4 T cells. Representative image of one of three independent experiments. The relative protein expression levels were quantified by densitometry and normalized to the control sample **(B)**. **(C)** Bar graph representing the relative expression levels of *CARKL* mRNA in WT CD4 T cells activated for 1, 2, 3 days or left non-activated. **(D)** CARKL protein concentration in naïve and activated CD4 T cells, retrieved from the Immunological Proteomic Resource. **(E)** qPCR analysis showing the expression of the glucose transporters *Glut1*, *Glut2*, *Glut3* and *Glut4* in WT and CARKLtm CD4 T cells upon activation. Data are presented as mean ± standard deviation (SD) from at least three independent experiments. *P < 0.05, ***P < 0.001, ****P < 0.0001 by two-sided paired *t*-test.

We investigated the expression of CARKL in CD4 T cells derived from both wild-type (WT) and transgenic (CARKLtm) mice. As expected, our data revealed a constitutive overexpression of CARKL in CD4 T cells from CARKLtm mice compared to WT CD4 T cells under both basal conditions and following activation, as depicted in Fig. 1A and B. Intriguingly, we noted a significant downregulation of CARKL in activated WT CD4 T cells when compared with their non-activated counterpart. The reduction in CARKL expression upon activation of CD4 T cells was further validated at the mRNA level in a time-dependent fashion (Fig. 1C). The Immunological Proteomic Resource (ImmPRes) confirmed such downregulation upon activation of murine CD4 T cells (Fig. 1D) (ImmPRes, Retrieved from http://immpres.co.uk/). A similar regulation was observed in a human CD4 T cell database (Supplementary Fig. S1) (The Human Protein Atlas, Retrieved from https://www.proteinatlas.org/). This temporal downregulation of *Carkl* mRNA expression provides insight into the dynamic regulation of CARKL in response to cellular activation.

T-cell activation is associated with a significant increase in metabolic demands, with enhanced glucose uptake playing a pivotal role in providing the necessary energy and resources for the activated T cells. This process is facilitated by the upregulation of specific glucose transporters (Glut 1–4) on the cell surface which enables efficient glucose uptake and utilization to support the metabolic activities required for T cell activation and function [19]. We investigated the mRNA expression of these transporters in WT and CARKLtm CD4 T cells upon activation. Our analysis revealed no significant differences in the expression levels of these transporters between WT and CARKLtm CD4 T cells (Fig. 1E), implying that alterations in CARKL expression are not associated with modifications in the gene expression of these transporters.

Notably, when comparing CD4 T-cell populations in WT versus CARKLtm at the steady state, we did not observe any major difference (Supplementary Fig. S2).

In conclusion, CARKL is constitutively overexpressed in transgenic CD4 T cells, while its expression is dynamically downregulated upon activation in wild-type cells, but this modulation does not impact glucose transporter expression or alter steady-state CD4 T-cell populations.

### CARKL overexpression results in a slight decrease in glycolysis

**Figure 2.**
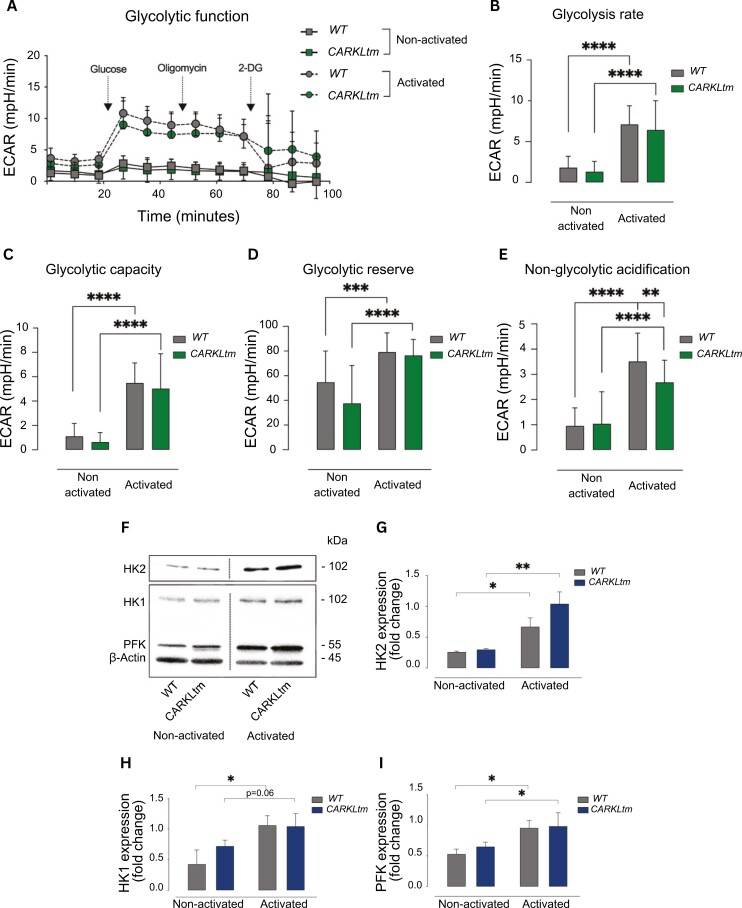
**Changes in CARKL expression results in a slight decrease in glycolysis. (A)** Graph showing the Extracellular Acidification Rate (ECAR) of WT and CARKLtm CD4 T cells before and after activation, as measured by the Seahorse XFe analyzer. Sequential injections of glucose (time point 1), oligomycin (time point 2), and 2-deoxyglucose (2-DG, time point 3) were performed to assess the glycolytic function. **(B)** Bar graph illustrating the baseline glycolysis rate in WT and CARKLtm CD4 T cells before and after activation. **(C)** Maximum ECAR achieved following oligomycin injection, indicating the glycolytic capacity of WT and CARKLtm CD4 T cells. **(D)** Difference between the maximum ECAR after oligomycin injection and the baseline ECAR before glucose injection, representing the glycolytic reserve. **(E)** ECAR measured after 2-DG injection, reflecting non-glycolytic acidification in WT and CARKLtm CD4 T cells. **(F)** Immunoblot showing HK2, HK1, PFK, and β-actin loading control protein levels in WT and CARKLtm CD4 T cells before and after activation. Representative image of one of three independent experiments. The relative protein expression levels were quantified by densitometry and normalized to the control sample **(G-I)**. Data are presented as mean ± standard deviation (SD) from at least three independent experiments. *P < 0.05, **P< 0.01, ***P < 0.001, ****P < 0.0001 by two-sided paired t-test.

To assess the glycolytic function and capacity of WT and CARKLtm CD4 T cells, before and after activation, the glycolysis stress test was performed using the Seahorse Xfe analyzer. Cells were subjected to sequential injections of glucose, oligomycin, and 2-DG, and ECAR was measured to evaluate glycolytic flux (Fig. 2A).

The results of the glyco stress test revealed notable differences between non-activated and activated WT and CARKLtm CD4 T cells. When glucose was injected, activated CD4 T cells, both WT and CARKLtm, displayed a significant rise in ECAR in comparison to non-activated T cells. Further analysis of the glyco stress test parameters demonstrated that activated WT and CARKLtm CD4 T cells exhibited comparable enhancements in glycolysis, glycolytic capacity, and glycolytic reserve when compared with non-activated cells, as depicted in Fig. 2B–D. Activated CARKLtm CD4 T cells exhibited lower levels of non-glycolytic acidification compared to activated WT T cells (Fig. 2E).

To further investigate the effects of CARKL overexpression on glycolysis, we also looked at the expression levels of key glycolytic enzymes, including HK1, HK2, and PFK (Fig. 2F–H). Notably, following activation, the expression of these enzymes increased in both CARKLtm and WT CD4 T cells.

Overall, despite similar increases in glycolytic enzyme expression after activation, CARKLtm CD4 T cells show reduced ECAR and lower non-glycolytic acidification compared to WT cells, indicating that CARKL overexpression impacts specific aspects of glycolytic function.

### CARKL-overexpressing T cells show impaired respiratory function

**Figure 3.**
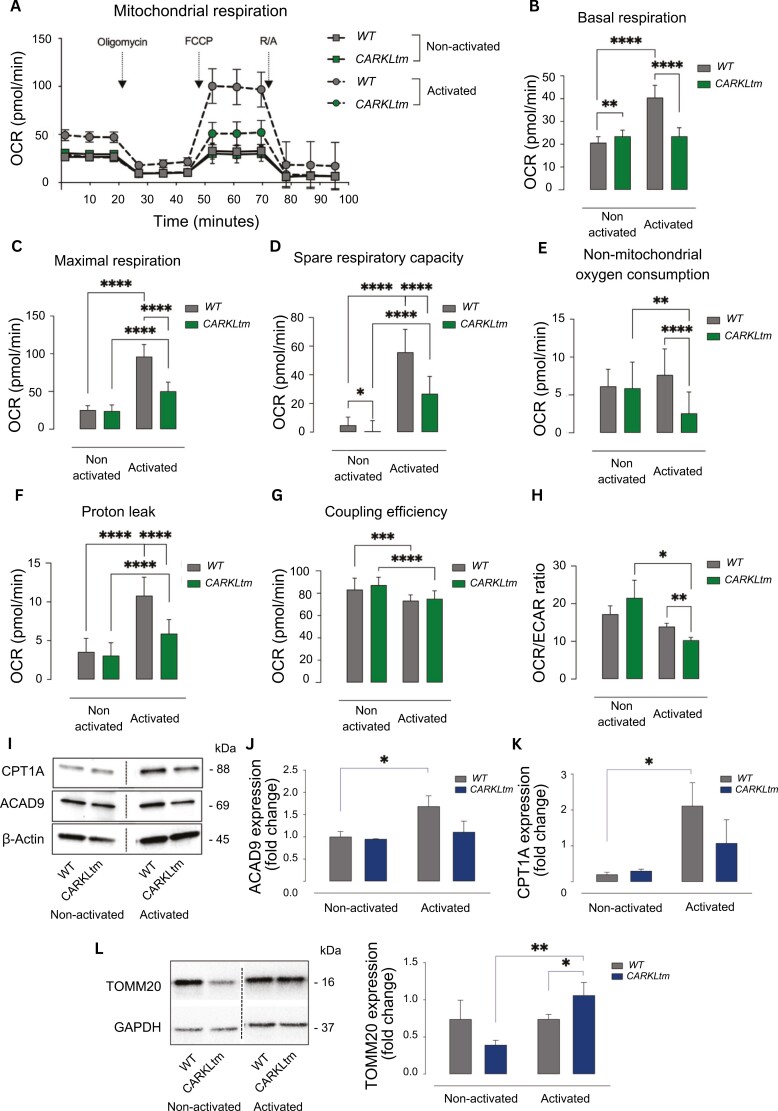
**CARKL overexpression impairs mitochondrial function and fatty acid oxidation in CARKLtm CD4 T cells upon activation. (A)** Representative graph showing the Oxygen Consumption Rate (OCR) of WT and CARKLtm CD4 T cells before and after activation, as measured by the Seahorse XFe analyzer. Sequential injections of oligomycin (time point 1), Carbonyl cyanide 4-(trifluoromethoxy)phenylhydrazone (FCCP, time point 2), and a combination of rotenone and antimycin A (time point 3) were performed to evaluate mitochondrial function. **(B)** Bar graph depicting the basal respiration rate of WT and CARKLtm CD4 T cells before and after activation. **(C)** Maximum OCR achieved after FCCP injection, indicating the maximal respiration capacity of WT and CARKLtm CD4 T cells. **(D)** Difference between maximal OCR after FCCP injection and basal OCR, reflecting the spare respiratory capacity. **(E)** OCR measured after rotenone and antimycin A injection, indicating non-mitochondrial oxygen consumption. **(F)** Bar graph comparing the proton leak in WT and CARKLtm CD4 T cells before and after activation, as derived from the OCR measurements after oligomycin injection. **(G)** Coupling efficiency, calculated as the proportion of basal respiration used for ATP production. **(H)** Ratio of basal OCR over basal ECAR in WT and CARKLtm CD4 T cells before and after activation. **(I)** Immunoblot showing CPT1A, ACAD9, and β-actin loading control protein levels in WT and CARKLtm CD4 T cells before and after activation. Representative image of one of three independent experiments. The relative protein expression levels were quantified by densitometry and normalized to the control sample **(J, K)**. **(L)** Immunoblot and quantification of TOMM20 and GAPDH loading control protein levels in non-activated and activated WT and CARKLtm CD4 T cells. Data are presented as mean ± standard deviation (SD) from at least three independent experiments. *P < 0.05, **P < 0.01, ***P < 0.001, ****P < 0.0001 by two-sided paired *t*-test.

To understand the metabolic energy profile and mitochondrial function of both non-activated and activated WT and CARKLtm CD4 T cells, the mitochondrial stress test was conducted using the Seahorse XFe analyzer Key parameters of mitochondrial function were assessed by tracking OCR (Fig. 3A), while sequentially introducing compounds that target distinct components of the mitochondrial electron transport chain. These compounds included oligomycin, which inhibits ATP synthase (complex V); FCCP, known to dissipate the proton gradient and disrupt the mitochondrial membrane potential, resulting in maximum oxygen consumption by complex IV; and a combination of rotenone and antimycin A, which inhibit complex I and complex III, respectively, effectively halting mitochondrial respiration [20, 21].

Cell activation was able to induce a significant increase in basal respiration only in WT CD4 T cells (Fig. 3B). Upon injection of oligomycin, a decrease in OCR was observed in both WT and CARKLtm CD4 T cells. The subsequent addition of FCCP led to a higher maximal OCR in WT CD4 T cells, reflecting the significantly reduced maximal respiration (Fig. 3C) and spare respiratory capacity (Fig. 3D) of CARKLtm CD4 T cells.

Following treatment with antimycin A/rotenone, the decrease in OCR was higher in CARKLtm CD4 T cells, signifying that the non-mitochondrial oxygen consumption rate is reduced compared to WT T cells (Fig. 3E), as well as the proton leak (Fig. 3F). No significant difference was observed in terms of coupling efficiency between WT and CARKLtm CD4 T cells (Fig. 3G).

Overall, the Seahorse results suggest that the overexpression of CARKL in activated CD4 T cells leads to discernible alterations in mitochondrial respiration parameters compared to the WT counterpart, characterized by a reduced OCR (Fig. 3H).

During fatty acid oxidation, the reduced forms of NAD and FADH2 transfer their electrons to the OXPHOS complexes. As a result, these pathways are interconnected biochemically and share substrates [22]. For this reason, we investigated the effects of CARKL overexpression on FAO enzymes (Fig. 3I). ACAD9 plays a crucial role in FAO by functioning as an acyl-CoA dehydrogenase enzyme within the mitochondrial matrix. Specifically, ACAD9 catalyzes the first step in the β-oxidation pathway by aiding in the breakdown of long-chain fatty acids into acetyl-CoA units. In addition to its role in FAO, ACAD9 has been implicated in the regulation of complex I of the electron transport chain in mitochondria. Notably, our observations revealed that the activation of WT CD4 T cells led to an upregulation in the expression of ACAD9, whereas T cells overexpressing CARKL did not exhibit a similar increase in ACAD9 expression levels (Fig. 3J). We also analyzed the expression of CPT1A, the gatekeeper enzyme that controls the entry of long-chain fatty acids into the mitochondria for subsequent breakdown. Similar to ACAD9, CPT1A expression was increased in WT T cells and not changed in CARKLtm T cells upon activation (Fig. 3K).

To gain further insights into the mitochondrial impact of CARKL overexpression in CD4 T cells, we examined the expression levels of TOMM20 (a well-established surrogate of mitochondrial mass) [23, 24]. We observed that TOMM20 expression was significantly increased in CARKLtm CD4 T cells following activation compared to WT cells (Fig. 3L), suggesting that decreased mitochondrial respiration in CARKLtm CD4 T cells does not occur due to reduced mitochondrial contents.

In conclusion, CARKL overexpression in CD4 T cells leads to significant alterations in mitochondrial function, including reduced maximal respiration, spare respiratory capacity, and fatty acid oxidation enzyme expression, highlighting a distinct metabolic profile compared to WT cells.

### CARKL overexpression leads to impaired energetic responses in T cells

**Figure 4.**
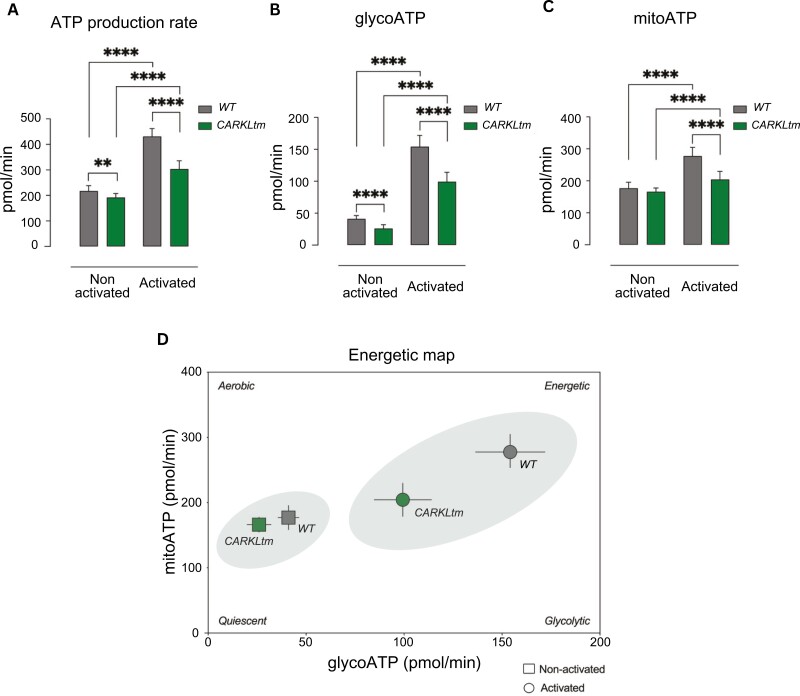
**Impaired ATP production in activated CARKLtm CD4 T cells assessed via XF Real-Time ATP Rate Assay. (A)** Bar graph showing the total ATP production rate in WT and CARKLtm CD4 T cells before and after activation, as measured by the XF Real-Time ATP Rate Assay. **(B)** Rate of ATP production from glycolysis in non-activated and activated WT and CARKLtm CD4 T cells. **(C)** Rrate of ATP production from oxidative phosphorylation (OXPHOS) in WT and CARKLtm CD4 T cells before and after activation. **(D)** Qualitative depiction of the cellular energy status of WT and CARKLtm CD4 T cells before and after activation. Data are presented as mean ± standard deviation (SD) from at least three independent experiments. **P < 0.01, ****P < 0.0001 by two-sided paired *t*-test.

To detect the total ATP production rates, serial additions of oligomycin and rotenone plus antimycin A were automatically and stepwise performed, during an XF Real-Time ATP Rate Assay. This metabolic assay allowed us to evaluate the amount of ATP produced by OXPHOS and glycolysis, which represent the two main metabolic pathways responsible for ATP production in mammalian cells. We analyzed the total cellular ATP production rate as well as the fractional contribution from glycolysis and oxidative phosphorylation, simultaneously. The assay revealed that total ATP production was significantly reduced in CARKLtm CD4 T cells as compared to WT cells, both before and after activation (Fig. 4A) with glycoATP production being affected (although the reduction in ECAR seen in the glyco stress test was small) before and after activation (Fig. 4B), and mitoATP production rate only after activation (Fig. 4C). The energy mapping analysis yielded a qualitative depiction of the cellular energy status (Figure 4D). Notably, prior to activation, both WT CD4 T cells and CARKLtm CD4 T cells displayed a quiescent state characterized by minimal glycolytic activity, relying predominantly on OXPHOS for energy production. Upon activation, a notable shift in cellular energy state was observed in WT CD4 T cells, marked by increased glycolysis and OXPHOS activity. However, CARKLtm T cells exhibited a notable deficiency in OXPHOS compared to WT T cells, thus rendering them less energetically proficient upon activation.

These findings suggest that CARKL overexpression impairs the ability of CD4 T cells to efficiently shift their energy metabolism upon activation, resulting in decreased total ATP production and an overall diminished metabolic capacity compared to WT cells.

### CARKL modulates the inflammatory profile of T cells

**Figure 5.**
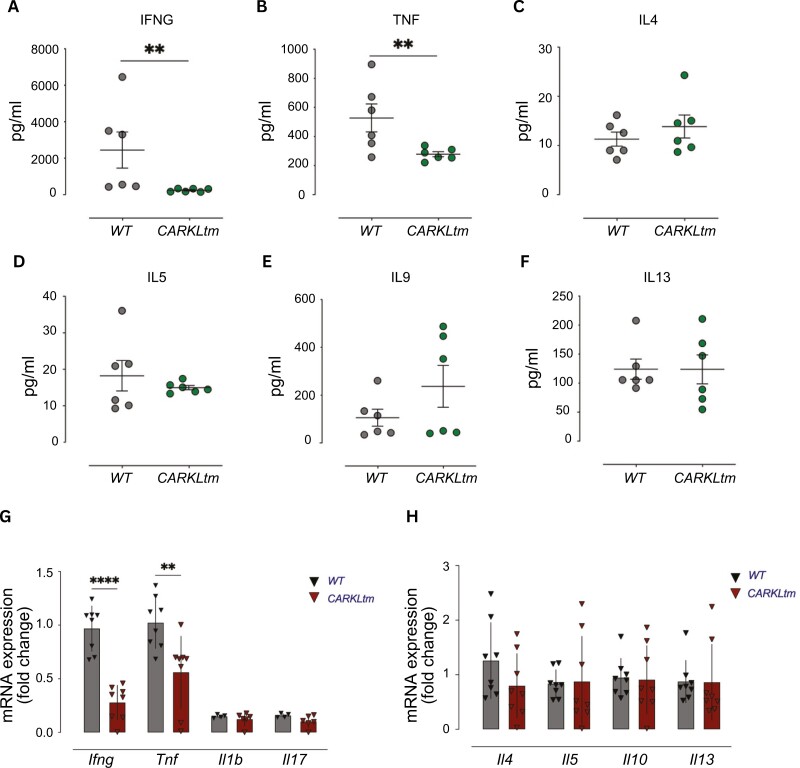
**CARKL overexpression alters inflammatory cytokine profile in CD4 T cells without affecting Th2 cytokine production.** Scatter plots showing the levels of IFNG **(A)**, TNF **(B)**, IL4 **(C)**, IL5 **(D)**, IL9 **(E)**, and IL13 **(F)** produced by WT and CARKLtm CD4 T cells before and after activation. Data are presented as mean ± SD from three independent experiments. **(G, H)** Scatter plots showing the mRNA expression levels of pro-inflammatory and immuno-modulatory cytokines as assessed by qRT-PCR in WT and CARKLtm CD4 T cells before and after activation. Statistical significance was determined using two-sided paired *t*-test, where **P < 0.01, ****P < 0.0001.

We next assessed whether the metabolic reshaping caused by CARKL overexpression (Figs. 3–4) accompanies changes in the CD4 T-cell effector functions, focusing on cytokine secretion and migration. We observed a marked decrease in the production of the inflammatory cytokines IFNG and TNF in CARKLtm as compared to WT CD4 T cells (Fig. 5A and B). The evaluation was conducted using a cytometric bead array assay with the BioLegend LEGENDplex Mouse Th Cytokine Panel. These two cytokines are predominantly secreted by Th1 T cells. Interestingly, the production of other cytokines associated with a Th2 T cell phenotype, such as IL4, IL5, IL9, and IL13, was similar between CARKLtm and WT CD4 T cells (Fig. 5C–F). These findings were corroborated by mRNA analysis, demonstrating a significant decrease in the gene expression of *Ifng* and *Tnf* in CARKLtm CD4 T cells compared to WT CD4 T cells (Fig. 5G). In contrast, the expression of *Il1b* and *Il17* (Fig. 5G), as well as *Il4, Il5, Il10,* and *Il13* (Fig. 5H) remained unaltered by CARKL overexpression. The observed alteration in the production of specific Th1 cytokines supports CARKL’s role in modulating the inflammatory profile of T cells.

In summary, overexpression of CARKL in CD4 T cells leads to a selective decrease in the production of Th1-associated cytokines, IFNG and TNF, without affecting Th2 cytokines. This suggests that CARKL plays a role in modulating the inflammatory response of T cells, particularly by influencing Th1 cytokine secretion.

### CARKL reduces CXCR3 expression on T cells, impairing cell motility

To explore the influence of CARKL overexpression on T-cell motility, we performed Transwell migration assays to examine the chemotaxis of CD4 T cells at different time points (2, 4, and 6 hours) following exposure to various chemokines. Given the distinct migratory characteristics exhibited by different T-cell subsets, we investigated the effects of several chemokines, including CXCL10, CCL22, and CCL19/21, which play roles in trafficking of Th1, Th2, and regulatory T cells, respectively.

**Figure 6.**
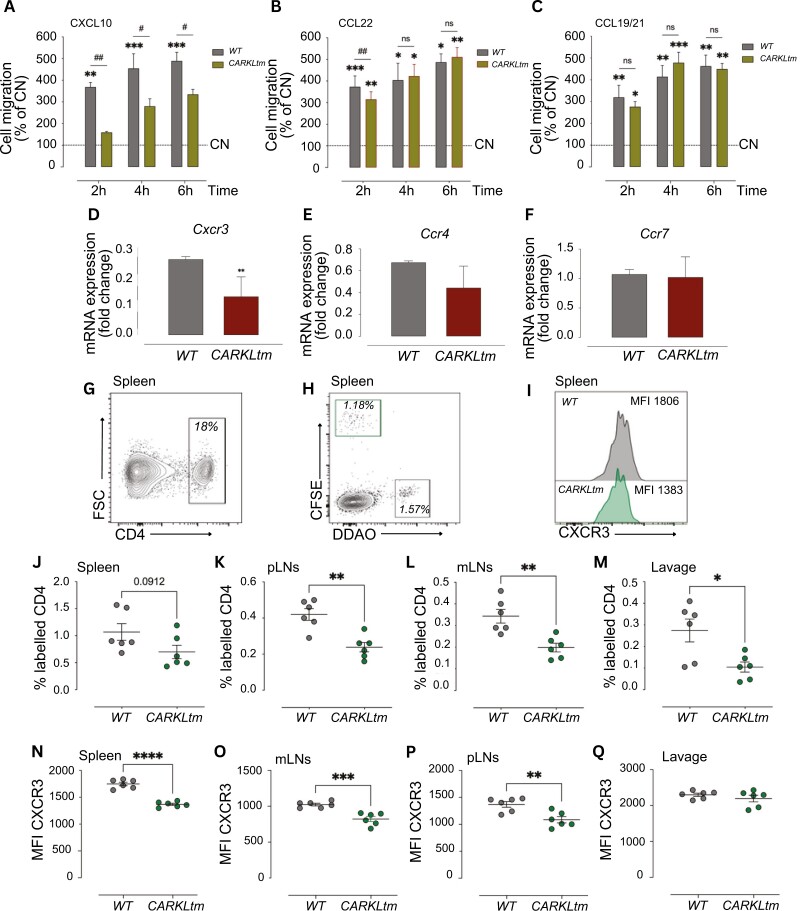
**Chemokinesis towards Cxcl10 is specifically inhibited in *CARKLtm* CD4 T cells.** Bar graphs showing the migration of activated WT and CARKLtm CD4 T cells towards CXCL10 **(A)**, CCL22 **(B)** and CCL19/21 **(C)** at different time points (2, 4, and 6 hours). CN: control, no chemokine. **(D-F)** Bar graphs showing the mRNA expression levels of the chemokine receptors *Cxcr3*, *Ccr4* and *Ccr7* in activated WT and CARKLtm CD4 T cells. Data are presented as mean ± standard deviation (SD) from three independent experiments. Representative flow cytometry plots showing the frequency of CD4 T-cell population identified in adoptive transfer recipient mice, which had received *in vitro* activated and labeled WT or CARKLtm CD4 T cells **(G)**, WT CFSE labeled and CARKLtm DDAO labeled identified within the CD4 T-cell population, 3 hours prior to receiving an intraperitoneal injection with CXCL10 (1200 ng/mouse) **(H)**, and histograms of CXCR3, indicating mean fluorescence intensity (MFI) gated from CFSE or DDAO labeled populations **(I)** from splenocytes isolated from recipient mouse. Scatter plots show the frequency of WT CFSE labeled and CARKLtm DDAO labeled cells within the CD4 T-cell population and MFI of CXCR3 from spleen **(J, N)**, pLN **(K, O)**, mLNs **(L, P),** and lavage **(M, Q)**. *P < 0.05, **P < 0.01, ***P < 0.001, ****P < 0.0001; #*P* < 0.05, ##*P* < 0.01, by two-sided paired *t*-test.

Our findings revealed that among the tested chemokines, only WT CD4 T cells displayed efficient migratory responses to CXCL10, whereas the motility of CARKLtm CD4 T cells in response to CXCL10 was markedly diminished (Fig. 6A). Interestingly, in the presence of CCL22 and CCL19/21, both WT and CARKLtm CD4 T cells exhibited increased motility compared to control T cells that were not exposed to any chemokine (Fig. 6B and C). These observations suggest that the impact of CARKL overexpression on T cell migration varies depending on the specific chemokine signaling pathways involved, highlighting the complex regulation of T-cell motility mediated by CARKL.

To further validate the hypothesis that CARKL overexpression contributes to the suppression of pro-inflammatory CD4 T-cell responses, we conducted an analysis of chemokine receptor expression in WT and CARKLtm CD4 T cells. Our investigation revealed that CARKL overexpression led to a decreased gene expression of *Cxcr3* (the CXCL10 receptor) in T cells, as illustrated in Fig. 6D. In contrast, the gene expression levels of *Ccr4* and *Ccr7* were not notably influenced by CARKL modulation (Fig. 6E and F). This differential regulation of chemokine receptor expression accounts for the distinct migratory capabilities of T cells in response to specific chemokines, providing insights into how CARKL selectively inhibits IFNγ-producing Th1 cells. These findings underscore the targeted impact of CARKL on modulating the immune responses associated with the pro-inflammatory Th1 phenotype.

To further corroborate our findings, we conducted a well-established model of lymphocyte recruitment to the inflamed peritoneum [25]. Consistent with transwell assays, CARKLtm CD4 T cells showed impaired trafficking to the inflamed peritoneum, *in vivo* (Fig. 6M). They also showed impaired trafficking to the other lymphoid tissues we assessed (Fig. 6J–L). In addition, CARKLtm CD4 T cells which had reached the peritoneum and lymphoid tissues showed a reduction in CXCR3 expression (Figs. 6N–Q).

In conclusion, CARKL overexpression impairs CD4 T-cell migration specifically in response to CXCL10, likely due to a reduction in CXCR3 expression, while migration towards other chemokines like CCL22 and CCL19/21 remains unaffected. This selective inhibition of CXCL10-mediated chemotaxis further supports CARKL’s role in modulating Th1-related pro-inflammatory immune responses.

## Discussion

Immunometabolism influences immune cell functions by regulating their metabolic pathways and responses to stimuli. PPP is a critical metabolic pathway that generates NADPH and pentose sugars, and CARKL, an enzyme involved in this pathway, has been shown to regulate macrophage polarization, suggesting it may be a potential therapeutic target for immune-related conditions [14, 26]. The results presented hereby shed light on the multifaceted role of CARKL in modulating CD4 T-cell metabolism, cytokine production, and migratory behavior. By employing a combination of Seahorse metabolic assays, gene expression analyses, and functional migration assays, we have delineated the impact of CARKL overexpression on T-cell function both *in vitro* and *in vivo*. These findings provide significant insights into how CARKL influences the immunometabolic landscape of CD4 T cells and their subsequent immune responses.

One of the key observations is the reduction in CARKL expression upon T-cell activation, indicating a regulatory role for CARKL in the metabolic state of CD4 T cells. This is further supported by the alterations in enzymatic activity related to the pentose phosphate pathway in CARKLtm CD4 T cells, suggesting a link between CARKL expression and metabolic changes in response to activation. These findings are in line with and supported by previous research on CARKL’s role in immune cell metabolism, particularly the study by Haschemi *et al*. [14], which elucidates CARKL’s influence on macrophage polarization through glucose metabolism. Haschemi *et al*. demonstrated that CARKL modulates macrophage polarization by regulating the PPP and thereby influencing the cell’s redox state and inflammatory profile. Our findings extend this understanding to CD4 T cells, showing that CARKL similarly affects critical metabolic pathways, including glycolysis and oxidative phosphorylation. Specifically, we observed impaired mitochondrial respiration in CARKLtm T cells, as evidenced by reduced basal and maximal respiration rates, and decreased spare respiratory capacity. This aligns with our observations of diminished ATP production from both glycolysis and OXPHOS in CARKLtm CD4 T cells, particularly upon activation. These metabolic deficiencies suggest that CARKL plays a crucial role in maintaining mitochondrial function and bioenergetic balance during T-cell activation. These findings are supported by previous research on metabolic regulation of immune cells which highlights the critical role of OXPHOS and mitochondrial function in T-cell activation and function [27–29]. Our results demonstrate that the diminished OXPHOS activity is associated with a reduced expression of FAO enzymes (CPT1A and ACAD9). This aligns with previous findings that spare respiratory capacity is associated with an enhanced capacity for FAO and increased expression of CPT1 [29, 30]. ACAD9 is also implicated in the assembly and stabilization of complex I of the ETC [31]. Complex I, or NADH:ubiquinone oxidoreductase, is the first enzyme complex in the mitochondrial respiratory chain and plays a pivotal role in establishing the proton gradient across the inner mitochondrial membrane, which drives ATP synthesis. Alterations in ACAD9 expression can lead to impaired complex I function, resulting in reduced electron transport and diminished ATP production [31]. The reduced expression of ACAD9 in CARKLtm cells likely contributes to their impaired OXPHOS. Reduced OXPHOS, coupled with the increased TOMM20 expression (a well-established marker of mitochondrial mass) observed in CARKLtm CD4 T cells upon activation, could also suggest a scenario of mitochondrial dysfunction. In response to mitochondrial damage or reduced respiratory chain efficiency, mitochondrial contents may adapt to compensate for loss of function and maintain energy homeostasis. Interestingly, despite the overall reduction in ATP production, the glycolysis stress test indicated that the glycolytic capacity and glycolytic reserve were only slightly reduced in CARKLtm CD4 T cells. This implies that while CARKL affects overall energy production, it does not drastically disrupt the glycolytic pathway.

CARKL overexpression significantly modulates the cytokine production profile of CD4 T cells. CARKLtm CD4 T cells showed a marked decrease in the production of the pro-inflammatory cytokines IFNγ and TNFα, both at the protein and mRNA levels. This reduction was specific to Th1-type responses, as the production of Th2-associated cytokines (IL4, IL5, IL9, and IL13) remained unaffected. These findings suggest that CARKL selectively suppresses Th1 responses, potentially through metabolic reprogramming that disfavors the bioenergetic demands of pro-inflammatory cytokine production. The observed decrease in pro-inflammatory cytokine production could be attributed to the impaired mitochondrial function in CARKLtm T cells. Efficient mitochondrial respiration is known to support the metabolic needs of activated T cells, particularly for the production of cytokines such as IFNγ and TNFα, further underscoring the critical role of mitochondrial respiration in T-cell function [27, 32, 33]. The compromised mitochondrial function in CARKLtm CD4 T cells likely hinders their ability to sustain such high-energy processes, thereby skewing their cytokine production profile. The role of CARKL in suppressing pro-inflammatory cytokines such as IFNγ and TNFα in CD4 T cells aligns with Haschemi *et al*.’s findings where CARKL downregulated pro-inflammatory cytokine production in macrophages [14]. Both studies highlight CARKL’s function in skewing immune responses towards a less inflammatory state. This suggests a conserved mechanism where CARKL modulates immune cell function by altering metabolic pathways and cytokine profiles, underscoring its potential as a therapeutic target in inflammatory diseases. Furthermore, it has been reported that overexpression of CARKL suppresses the mRNA expression of *G6PD*, the key enzyme in the pentose phosphate pathway. This overexpression also leads to a marked reduction in cell proliferation, ROS production, and IL1β secretion [18].

Our migration assays demonstrate that CARKL overexpression affects T-cell motility in response to specific chemokines. CARKLtm CD4 T cells showed significantly reduced migration in response to CXCL10, a chemokine critical for the trafficking of Th1 cells [34–36]. This impaired migratory response correlates with the decreased expression of CXCR3, the receptor for CXCL10, in CARKLtm T cells and highlights the role of CARKL in regulating chemokine receptor expression and function. In contrast, migration in response to CCL22 and CCL19/21, which are important for Th2 and regulatory T-cell trafficking, respectively, was not significantly affected by CARKL overexpression. This differential impact on chemokine-mediated migration underscores the selective influence of CARKL on Th1 cell behavior, further supporting its role in modulating pro-inflammatory T cell responses. The *in vivo* lymphocyte recruitment assays corroborate our *in vitro* findings, showing that CARKLtm CD4 T cells have impaired trafficking to inflamed peritoneum and other lymphoid tissues. This reduced migratory capacity *in vivo* is consistent with the decreased CXCR3 expression and impaired chemotaxis in response to CXCL10 observed *in vitro*. These results suggest that CARKL overexpression not only affects T-cell function at a cellular level but also has significant, systemic implications for immune responses *in vivo*, particularly in contexts requiring efficient Th1 cell migration and function. These results suggest that CARKL expression can be a rate-limiting step for balancing metabolic intermediates of the PPP, and its modulation can determine the phenotype of activated T cells.

Overall, our study reveals that CARKL plays a critical role in orchestrating the metabolic and functional dynamics of CD4 T cells. By modulating mitochondrial function, cytokine production, and migratory behavior, CARKL emerges as a key regulator of T-cell-mediated immune responses. These insights provide a deeper understanding of the metabolic regulation of T-cell function and highlight potential therapeutic targets for modulating immune responses in inflammatory and autoimmune diseases. Further research is warranted to explore the precise mechanisms through which CARKL influences T-cell metabolism and to investigate its potential as a therapeutic target in immune-related disorders.

## Supplementary Material

kyae016_suppl_Supplementary_Figure_S1

kyae016_suppl_Supplementary_Figure_S2

## Data Availability

All the data underlying this article are included in the manuscript. Data files will be made available upon reasonable request to the corresponding authors.

## Abbreviations

2-DG: 2-Deoxy-d-glucose
CCL19/21: Chemokine (C-C motif) ligand 19/21
CCL22: Chemokine (C-C motif) ligand 22
CCR4: C-C chemokine receptor type 4
CCR7: C-C chemokine receptor type 7
CXCL10: Chemokine (C-X-C motif) ligand 10
CXCR3: C-X-C chemokine receptor type 3
ECAR: Extracellular acidification rate
ETC: Electron transport chain
F6P: Fructose-6-phosphate
FCCP: Carbonyl cyanide-p-trifluoromethoxyphenylhydrazone
G3P: Glyceraldehyde 3-phosphate
G6P: Glucose-6-phosphate
G6PD: Glucose-6-phosphate dehydrogenase
HK1: Hexokinase 1
HK2: Hexokinase 2
LPS: Lipopolysaccharide
NADPH: Nicotinamide adenine dinucleotide phosphate
OCR: Oxygen consumption rate
PFK: Phosphofructokinase
R5P: Ribose-5-phosphate
Ru5P: Ribulose-5-phosphate
S7P: Sedoheptulose-7-phosphate
X5P: Xylulose-5-phosphate
