# Supplementary figures and images for: The sedoheptulose kinase CARKL controls T-cell cytokine outputs and migration by promoting metabolic reprogramming
Source: Discov Immunol. 2024 Nov 19;3(1):kyae016. doi: 10.1093/discim/kyae016 (PMC11635167; doi:10.1093/discim/kyae016)

*CARKL*

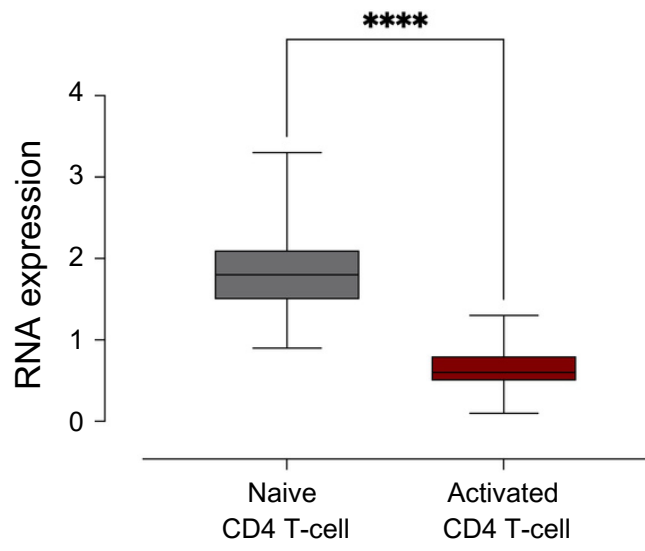

Supplement: kyae016_suppl_Supplementary_Figure_S1 [file kyae016_suppl_supplementary_figure_s1.pdf]

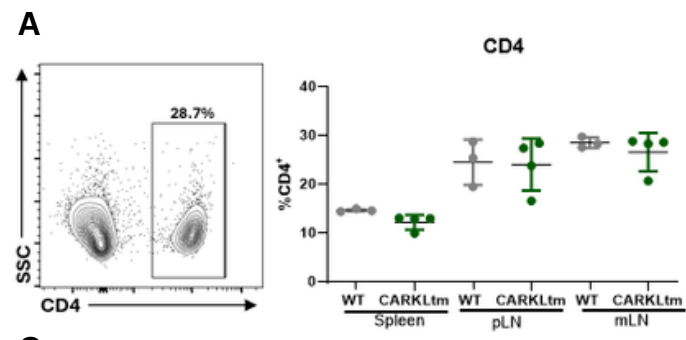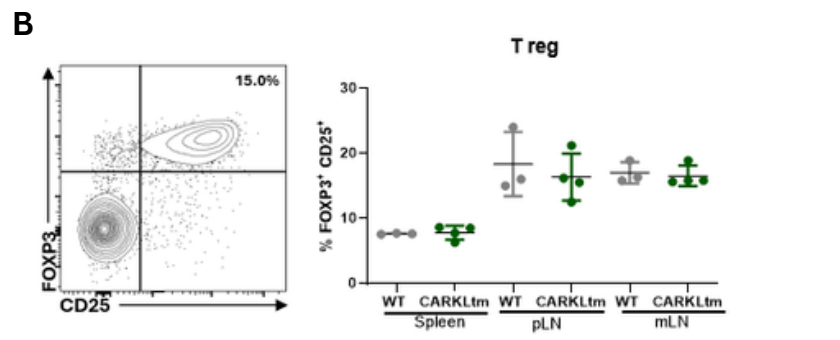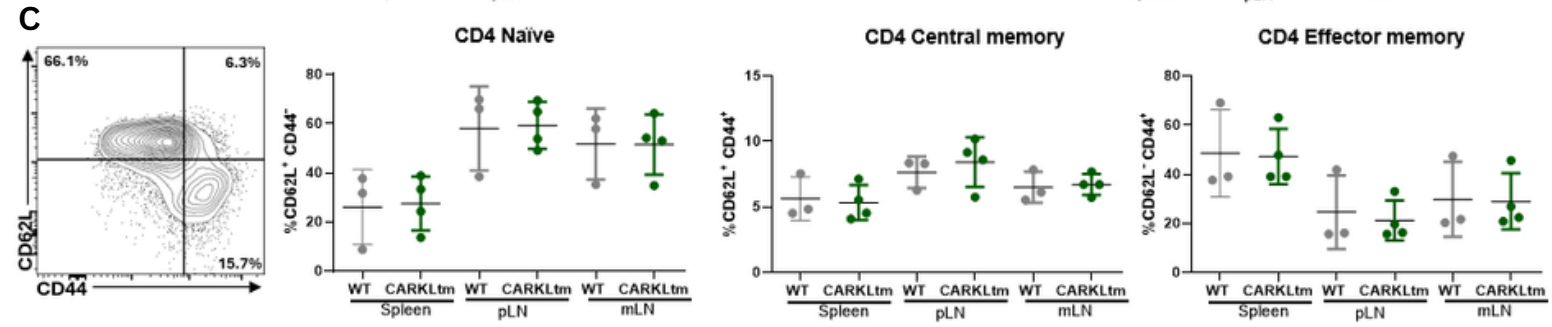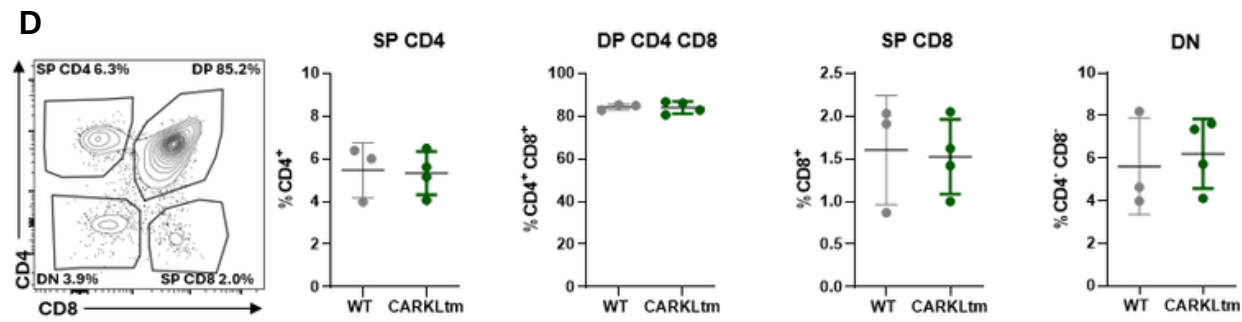

Supplement: kyae016_suppl_Supplementary_Figure_S2 [file kyae016_suppl_supplementary_figure_s2.pdf]
